# Equity in household spending on alcoholic beverages in South Africa: assessing changes between 1995 and 2011

**DOI:** 10.1186/s12939-019-0985-3

**Published:** 2019-05-28

**Authors:** Mayara Fontes Marx, Leslie London, Nadine Harker, John E. Ataguba

**Affiliations:** 1 Health Economics Department, School of Public Health and Family Medicine, Health Science Faculty, University of Cape Town, Observatory, Cape Town, 7925 South Africa; 2 Health and Human Rights Programme, School of Public Health and Family Medicine, Health Science Faculty, University of Cape Town, Observatory, Cape Town, 7925 South Africa; 3 Alcohol Tobacco and Other Drug Research Unit, South African Medical Research Council, Francie van Zijl Drive, Parow Valley, Cape Town, South Africa

**Keywords:** Alcoholic beverages, Progressivity, Spending, Alcohol consumption, Alcohol policy

## Abstract

**Background:**

Globally, alcohol consumption accounts for a substantial burden of disease, which translates into high social and economic costs. To address this burden, several policies (e.g. age and trading hour restrictions, increasing alcohol taxation) were implemented. Despite the existence of these policies evidence shows that alcohol misuse and alcohol-related harms have increased in South Africa over recent years. The objective of this paper is to assess progressivity and the changes in progressivity of alcohol expenditure at the household level in South Africa using datasets that span 15 years.

**Methods:**

Data come from the 1995, 2000, 2005/06 and 2010/11 South Africa Income Expenditure Survey. Distribution of spending on alcoholic beverages were analyzed using standard methodologies. Changes in progressivity between 1995 and 2000, and between 2005/06 and 2010/11 were also assessed using the Kakwani index.

**Results:**

Alcohol spending was regressive between 1995 and 2011 as the fraction of poorer households’ expenditure spent on alcohol beverage exceeds that for the richest households. Also, the difference in Kakwani indexes of progressivity indicates that spending on alcoholic beverages has become less regressive between the same time periods.

**Conclusion:**

The results show no evidence that alcohol policy including taxation increased regressivity. Thus, there is an opportunity to further reduce the regressivity using coherent alcohol policies. This paper concludes that there is a need for further research to unpack why alcohol spending became less regressive over the years that goes beyond just looking at changes in the distribution of alcohol expenditure.

## Background

Globally, alcohol is responsible for 5.9% of deaths and 5.1% of the burden of disease and injury, which is equivalent to 139 million disability-adjusted life years lost (DALYs) due to premature death and the time lost due to time lived in less than full health [1]. Worldwide, alcohol is also considered one of the top three risk factors for disease and injury [1]. To address this burden and to reduce alcohol-related harm, alcohol policies have been used extensively by governments [2]. Two classes of alcohol policies can be distinguished — allocative and regulatory [2]. Allocative alcohol policies are those that assist in raising resources (e.g. funding) to a group or organization to achieve public objectives. Cases where the government provides funding for education to prevent or reduce harm from alcohol in schools form an example of allocative alcohol policy [2]. Regulatory alcohol policies are designed to influence or control individuals and organizations’ actions, behaviors, and decisions, for instance, price control, taxation, restrictions on alcohol sales, alcohol advertising and consumption [2].

Internationally, regulatory alcohol policies that aim to reduce affordability of alcohol beverages are widely used and are considered one of the most cost-effective strategies to decrease alcohol consumption and alcohol-related harms [3]. These price-base policy interventions are based on the premise that if the demand for alcohol is price elastic, then by increasing alcohol prices, consumers will consume less alcohol due to budget constraints. Nevertheless, the literature is mixed on the real size of the impact of alcohol price elasticity on alcohol consumption as different types of alcoholic beverages are closely related substitutes and might have different price elasticities [2]. For example, an increase in price of alcohol due to regulatory policy, e.g. tax, can result in a deadweight loss or an excess burden of taxation as consumers make inefficient choices like substituting their alcohol consumption for a cheaper, unregulated and lower quality version such as homemade brews [4] to avoid taxation. Thus, overall spending on alcohol might be reduced, but alcohol consumption and alcohol-related harm may not decline. Another example of unanticipated and undesirable effects of price increases is when consumers reallocate money that would be spent on some other household items to maintain the same level of alcohol consumption as before the increase in tax. This may contribute to further impoverishment of affected families with no beneficial impact on reducing alcohol consumption. Also, alcohol policies that aim to decrease affordability may not be equitable if alcohol taxes are regressive, meaning the poor pay proportionately more alcohol taxes than the rich when compared to their shares of income [5, 6].

In the last two decades in South Africa, alcohol pricing increases were implemented not only to raise revenue but also to decrease alcohol consumption and the negative impacts of alcohol misuse [7, 8]. Between 1994 and 2013, on average, alcohol tax rate increases have been higher than increases in the Consumer Price Index (CPI) [8]. For instance, between 1994 and 1995, malt beer, unfortified wine and spirits had a tax increase of 14, 24 and 10%, respectively while the CPI increased by 9%. While between 2011 and 2012, the equivalent increases were 10, 8 and 20%, respectively while the CPI increased by 6%. That means alcohol taxation in South Africa has been rising faster than the general prices levels (i.e. CPI). Nonetheless, compared to high-income countries, South African consumers pay far less tax on their alcohol. In South Africa, 20% of the final price of alcohol purchased off-premise comprises excise tax, while in Australia and New Zealand the percentage of excise tax off-premise were 30 and 37% of the final price, respectively. However, for middle-income countries the percentages are low- In Vietnam and St Kitts excise tax on alcohol purchased off-premise were between 7 and 8% of the final price [9].

While an increase in alcohol tax is expected to decrease alcohol consumption, evidence shows that alcohol misuse and alcohol-related harm in South Africa have increased in recent years. Cross-sectional analyses confirm an increase in current drinkers (defined as consuming alcohol in the past 12 months) from 15.8% in 2005 to 18.2% in 2008 and to 21.7% in 2012 [10]. Occasional heavy drinkers (defined in the study as the same as binge drinking or consuming 5 or more drinks for a man or consuming 4 or more drinks for women on a single occasion) increased from 9.8% in 2005 to 13.2% in 2012 [10].

Although alcohol pricing policy may appear to have no effect on decreasing overall alcohol consumption, one should consider that in the absence of these regulatory alcohol policies, alcohol misuse and alcohol-related harm in South Africa may have risen much more than it did. Other factors such as agressively marketing by the alcohol industry to attract new consumers (specially young people and women) and to normalize regular drinking [11] may have countered any effects arising from price increases.

With alcohol misuse and alcohol-related harms not expecting to slow down, many researchers are advocating for additional and tougher alcohol regulation and an increase in alcohol laws enforcement levels [11–14]. Thus, there is a need to understand how regulatory alcohol policies, especially exogenous tax increases that aim to reduce affordability affect spending on alcohol beverages at the household level. Thus, this paper assesses changes in the progressivity of spending on alcoholic beverages (i.e. how the share of such spending in total household income varies between richer and poorer households) in South Africa. This is done by comparing changes between 1995 and 2010/11. Specifically, changes between 1995 and 2000 are compared with changes between 2005/06 and 2010/11. As detailed below, the split in the comparison is motivated by similarities in the methodologies used for data collection between the1995 and 2000 period and between the 2005/06 and 2010/11 period but differences in methods between the two periods. To our knowledge, apart from a first attempt to assess progressivity in alcohol taxes in South Africa [6], this paper represents the first analysis of the progressivity and the changes in progressivity of alcohol expenditure at the household level in South Africa using datasets that span 15 years. The previous progressivity study [6] only looked at the effect of alcohol taxes. Thus, to fill up the gap, this paper looks at household expenditure on alcoholic beverages as many alcohol policies including tax will have significant effect on household spending. This analysis is useful in assessing, among other things, the impact of existing and future alcohol regulatory policies using price as a lever to reduce affordability of alcohol beverage over time.

## Method

### Data

This paper uses the Income and Expenditure Survey (IES) datasets compiled by Statistics South Africa, the national statistical authority. The IES is a national household survey conducted every five years. It contains data on household expenditure on different items including housing, transportation, health, education, food and beverages. The IES provides data on the amount households spent on specific types of alcoholic beverage (e.g. how much was spent on beer or wine in the last month). IES datasets are used to calculate the South African CPI and a range of other socio-economic indicators used in many different analyses [15, 16]. IES datasets were accessed through DataFirst.^1^

Table 1 summarizes the methodology including the survey methods used in each IES round. The IES data have a nationally representative sample of 29,595 households in 1995; 26,265 households in 2000; 21,144 households in 2005/06 and 25,328 households in 2010/11. The main difference between IES rounds is that the IES 1995 and 2000 used face-to-face recall recorded by interviews at a household visit while the IES 2005/06 and 2010/11 used a mix of face-to-face recall by interview and diary method (i.e. households are given a new diary every week for four weeks to record actual purchases). In total, every household in the IES 2005/06 and 2010/11 was visited five times (one visit for the main questionnaire and four visits for the weekly diaries). Due to changes in the IES survey methodology, alcohol expenditure patterns will be stratified by comparisons between (a) 1995 and 2000 and (b) 2005/06 and 2010/11. The earliest IES data, collected in 1990, will not be included in this analysis since its methodology and data collection process are not comparable with subsequent IES rounds; it only covers 12 major metro/urban areas and data for the “white” population group are not available (Table 1).

**Table 1:** Income Expenditure Survey (IES) dataset summary - 1990 to 2010/2011

	1990	1995	2000	2005/2006	2010/2011
Number of Observations (Households)	47,781	29,595	26,265	21,144	25,328
Geographic Coverage	Twelve major metro/urban areas. Leaves out small towns and rural areas.	National Coverage- metropolitan, urban and rural areas.	National coverage. Covered de jure household members.	National coverage. Covered all household members.	National coverage. Covered all household members.
Geographic Unit	Magisterial district	Magisterial district	Magisterial district	Province	Province
Data Collection	Face-to-face. Recall Method.	Face-to-face. Recall Method.	Face-to-face. Cases where the household requested to complete the questionnaire themselves and have the completed questionnaire collected at a second visit. Recall Method.	Face-to-face. Combination of recall and diary method. Five separate visits to collect the diaries and questionnaires.	Face-to-face. Combination of recall and diary method. Five separate visits to collect the diaries and questionnaires.
Questionnaires	2 questionnaires- Long and short.	Questionnaire has monthly (1–31 October 1995) and annual (October 1994–October 1995) expenditure sections. The monthly expenditure was multiplied by 12.	Interview the household head or a responsible adult.	Households were given diaries and required to record their daily purchases over a period of 4 weeks. Fieldworker administered the main questionnaire.	Households were given diaries and required to record their daily purchases over a period of 4 weeks. Fieldworker administered the main questionnaire.
Units of Analysis	Household and individuals	Household and individuals	Household and individuals	Household and individuals	Household and individuals
Limitations	There is no data file for the “white” population group. Recall Method.	Recall Method	Recall Method	No estimates at a municipal or district level.	No individual unit. No estimates at a municipal or district level.

Source: [17–21]

### Key variables and estimation strategy

Table 2 summarizes the key variables used in the analysis. Economic theory suggests that consumption, defined as resources consumed, is a preferable measure of living standards than income. This is because income can be saved and many surveys do not account for household production in its calculation [22]. Additionally, in developing countries income data are not reliable [23]. Thus, this paper uses household consumption expenditure (sometimes referred to as household expenditure in this paper) as a direct measure for living standards. Household consumption expenditure and all spending on alcohol beverages were adjusted by household size (Table 2). The 1995, 2000, 2005/06 and 2010/11 data were adjusted to 2016 prices using the South African CPI data. Then, the variables were converted into 2016 US dollars using the exchange rate R14.71 to the dollar. All data cleaning, exploration and analysis were done using Stata 12 statistical software [24].

**Table 2:** Description of key variables

Variables	Definition
Spirits (per capita)	Total annual spending on brandy, whisky, gin and other spirits (including liqueur) divided by the household size.
Beer (per capita)	Total annual spending on beer including lager and cider divided by the household size.
Sorghum (per capita)	Total annual spending on (pre-packed) and traditional beer divided by the household size.
Wine (per capita)	Total annual spending on table wines (including sparkling wine and juice/ wine mixtures), fortified wines (sherry and port.) and cooking wines divided by the household size.
Other (per capita)	Total annual spending on other alcohol divided by the household size.
Total expenditure on alcohol (per capita)	Total annual spending on spirits, beer, sorghum, wine and other divided by the household size.
Total household consumption expenditure (per capita)	Total annual spending on cost of housing, food, non- alcohol beverages, alcohol beverages, clothing and footwear, health services, recreation and entertainment and own production and consumption home grown products divided by the household size.

Note: Purchasing includes items and services purchased and consumed in cafes, restaurants, hotels, shebeens (defined as an informal unlicensed drinking place in a township), taverns. Purchased in shops, cafes, liquor outlets, formal or informal, but consumed elsewhere. Source: [17–21]

**Table 3:** Summary of measurements of effective progressivity of alcohol spending in South Africa

	Definition
Lorenz curve	The Lorenz curve assesses the degree of inequality in socio-economic status (SES) in South Africa.
Concentration curve	The concentration curve is the degree of inequality in alcohol expenditure between poor and wealthier households in South Africa.
The concentration index	The concentration index (*C*) is derived from the concentration curve. Its values can vary from − 1.0 (where all expenditure on alcoholic beverages is made by the poorest household) to + 1.0 (where all alcohol expenditure is made by the richest household).
The Gini index	The Gini index (G) is derived from the Lorenz curve. It corresponds to consumption expenditure inequality. It can vary from 0 (perfect equality in the distribution of consumption expenditure) to 1 (perfect inequality in the distribution of consumption expenditure).

Sources: [22, 25]

### Progressivity analysis

A progressivity framework borrowed from public sector economics, which has also been used in the assessment of progressivity in health financing [25, 26], was used in this analysis. Two approaches may be used to assess progressivity: structural and effective progressivity [25]. These were applied to estimate progressivity and the changes in progressivity of alcohol expenditure in South Africa.

### Structural progressivity analysis

Structural progressivity is usually assessed by comparing the distribution of spending on alcohol beverages as a percentage of total household consumption expenditure in each quintile of household consumption expenditure per capita [22]. This was assessed in Stata using a user-written -fia- command [25]. If the share of consumption expenditure on alcohol increases with the quintiles (i.e. households that are richer spend a greater share of consumption expenditure on alcohol), then spending on alcohol beverages is progressive. If the share of consumption expenditure on alcohol decreases with the quintiles (i.e. households that are richer spend a smaller share of consumption expenditure on alcohol), then spending on alcohol beverages is regressive.

### Effective progressivity analysis

The Kakwani index of progressivity [27] was used to assess effective progressivity of alcohol spending in South Africa. The Kakwani index is a well-known and widely used measure of progressivity. It compares the distribution of household consumption expenditure (using the Lorenz curve [Table 3]) with that of alcohol spending (using concentration curve [Table 3]).

Effective progressivity was also computed using the -fia- command [25]. The framework for effective progressivity has been detailed elsewhere [25]. In brief, the Kakwani index (*K*) for any alcoholic beverage was computed as the difference between the Gini index (*G*) of consumption expenditure and the concentration index (*C*) of expenditure on the specified alcoholic beverage.

1 $$ K=C-G $$

A progressive spending on alcoholic beverages occurs when *C* > *G*, while regressive spending occurs when *C* < *G*. Proportional spending on alcoholic beverages occurs when *C* = *G*. Numerically, the value of the Kakwani index can vary from − 2 (most regressive) to 1 (most progressive). A positive value (*K* > 0) means that alcohol expenditure is progressive as richer households spend proportionately more on alcoholic beverages than their share of consumption expenditure. A negative value (*K* < 0) represents the opposite [25].

Effective progressivity between two-time periods was calculated by the difference in the Kakwani index between two-time periods [26].

2 $$ \Delta  K={K}_t-{K}_{t-1} $$

From eq. 1,

3 $$ \Delta  K=\left({C}_t-{C}_{t-1}\right)-\left(, {G}_t,-,{G}_{t-1}\right) $$

4 $$ \Delta  K=\Delta  C-\Delta  G $$

The difference in the Kakwani indexes between the two-time periods (eq. 4) can result in a pro-poor ‘shift’ or a pro-rich ‘shift’. A pro-poor ‘shift’ occurs when the Kakwani index becomes more progressive or less regressive over time (e.g. *∆K* > 0; *∆C* > *∆G*); while a pro-rich ‘shift’ occurs if otherwise (i.e. *∆K* < 0; *∆C* < *∆G*) (see Ataguba [26] for additional progressivity or regressivity scenarios). The standard errors for differences in the indexes between time periods (e.g. *∆K* = *K*_*t*_ − *K*_*t* − 1_) were obtained using the bootstrap methods with 1000 resamples based on the full sampling structure (see also Hall and Wilson [28] for additional detail).

## Results

Overall, per capita alcohol spending in South Africa has increased by $0.33 in real terms, between 1995 and 2000; while it decreased by $1.87 in real terms, between 2005/05 and 2010/11 (Table 4). In absolute terms, as expected, richer households spend much more on alcohol than poorer households.

**Table 4:** Average annual alcohol expenditureª per capita by quintile in South Africa ($US), 1995–2011

	(a) 1995	(b) 2000	(c) = (b)- (a) 2000–1995	(d) 2005/06	(e) 2010/11	(f) = (e)-(d) 2010/11–2005/06
Poorest	0.25	0.21	− 0.04*	3.33	2.79	−0.54**
2nd Quintile	0.71	0.67	−0.04	9.19	8.52	−0.67
3rd Quintile	1.57	1.61	0.04	18.46	20.78	2.32*
4th Quintile	3.02	3.14	0.12	42.81	33.51	−9.30***
Richest	8.26	9.82	1.56***	71.61	70.46	−1.15
Total	2.76	3.09	0.33*	29.08	27.21	−1.87*

ªSpending on all alcoholic beverages. Significance levels are denoted as follows: ***1%, **5%, *10%

Note: Consumption expenditures are expressed in 2016 dollars. The averages are computed for the entire population (includes household consumption expenditure of drinkers and non-drinkers)

Average per capita spending on spirits decreased for all quintiles over the years (Table 5). For wine, average spending per capita is approximately the same between 1995 ($0.45) and 2000 ($0.40); while it increased by $0.59 in real terms, between 2005/06 and 2010/11. Average spending per capita on beer increased by $0.50 in real terms, between 1995 and 2000; while it decreased by $2.23 in real terms, between 2005/06 and 2010/11. Excluding the 4th quintile, where average spending per capita on sorghum decreased from $1.53 in 2005/06 to $1.21 in 2010/11, sorghum is the only alcoholic beverage that average spending per capita increased over the years and across quintiles. Overall, the poorest quintile has a pattern of decreasing average spending on specific alcohol beverages over the years. The richest quintile has increased average spending per capita on wine and sorghum over the years and decreased average spending per capita on spirits over the years. For beer, average spending per capita for the richest households increased between 1995 and 2000; while it decreased between 2005/06 and 2010/11.

**Table 5:** Average annual per capita consumption expenditure on specific alcohol beverages in South Africa ($US), 1995–2011

		Poorest	Q2	Q3	Q4	Richest	Total	Total Differenceª
Spirits	1995	0.03	0.10	0.27	0.67	2.75	0.76	
	2000	0.01	0.06	0.15	0.47	2.38	0.61	−0.15***
	2005/06	0.24	1.03	2.50	6.88	24.17	6.96	
	2010/11	0.20	0.68	2.46	5.50	21.38	6.04	−0.92**
Wine	1995	0.02	0.08	0.20	0.27	1.69	0.45	
	2000	0.01	0.04	0.09	0.15	1.70	0.40	−0.05**
	2005/06	0.43	0.73	0.88	2.70	13.11	3.57	
	2010/11	0.35	0.73	1.09	1.10	17.51	4.16	0.59*
Beer	1995	0.12	0.37	0.87	1.88	3.57	1.36	
	2000	0.09	0.39	1.05	2.25	5.51	1.86	0.50***
	2005/06	1.76	6.25	13.12	31.64	33.98	17.34	
	2010/11	1.40	5.36	14.95	25.67	28.19	15.11	−2.23**
Sorghum	1995	0.08	0.14	0.21	0.19	0.17	0.16	
	2000	0.09	0.18	0.31	0.27	0.20	0.21	0.05***
	2005/06	0.87	1.11	1.83	1.53	0.04	1.08	
	2010/11	0.78	1.63	2.16	1.21	3.36	1.83	0.75**

ªDifference in the total column. Significance levels are denoted as follows: ***1%, **5%, *10%

Note: Expenditures are expressed in 2016 dollars. The averages are computed for the entire population (includes household consumption expenditure of drinkers and non-drinkers)

### Assessing progressivity of spending on alcohol beverages in South Africa

#### Structural progressivity of alcohol expenditure in SA 1995 to 2011 results

The results in Table 6 show that overall alcohol spending as a share of total household consumption expenditure increased slightly by 0.03%, in absolute terms, between 1995 and 2000. However, this is not statistically significant. On the other hand, it decreased significantly by 0.19% between 2005/06 and 2010/11. The three middle quintiles spent the most on alcohol beverages as a share of their expenditure (particularly, the third quintile) at all time periods. Overall, there is an inverted J shape for the proportion of consumption expenditure spent on alcohol in South Africa. In this analysis, it is difficult to ascertain overall progressivity by only looking at the ratios in Table 6 as these may vary across the entire distribution of consumption expenditure. However, the indexes in Table 7 provide the extent of progressivity of alcohol spending.

**Table 6:** Proportion of consumption expenditure spent on alcoholª in South Africa, 1995–2011

	(a)	(b)	(c) = (b)-(a)	(d)	(e)	(f) = (e)-(d)
	1995	2000	2000–1995	2005/06	2010/11	2010/11–2005/06
Poorest	0.10%	0.09%	−0.01%	1.16%	0.82%	−0.34%*
2nd Quintile	0.12%	0.13%	0.01%	1.60%	1.20%	−0.40%*
3rd Quintile	0.13%	0.16%	0.03%	1.83%	1.63%	−0.20%
4th Quintile	0.11%	0.15%	0.04%	2.00%	1.26%	−0.74%***
Richest	0.08%	0.11%	0.03%	0.71%	0.63%	−0.08%
Total	0.09%	0.12%	0.03%	1.03%	0.84%	−0.19%**

ªSpending on all alcoholic beverages. Significance levels are denoted as follows: ***1%, **5%, *10%. The averages are computed for the entire population (includes household consumption expenditure of drinkers and non-drinkers)

**Table 7:** Progressivity of spending on specific alcohol beverages in South Africa, 1995–2010/11

	(a) 1995	(b) 2000	(c) = (b) -(a) ª 2000–1995	(d) 2005/06	(e) 2010/11	(g) = (d) - (e) ª 2010/11–2005/06
Gini index	0.652***	0.658***	0.006	0.664***	0.643***	−0.021***
	(0.0090)	(0.0095)	(0.0084)	(0.0207)	(0.0100)	(0.0108)
Concentration Index						
Total Alcohol Consumption	0.579***	0.602***	0.023***	0.497***	0.498***	0.001
	(0.0112)	(0.0125)	(0.0154)	(0.0172)	(0.0132)	(0.0228)
Spirits	0.691***	0.753***	0.062	0.664***	0.660***	−0.004
	(0.0131)	(0.0183)	(0.0189)	(0.0241)	(0.0172)	(0.0267)
Wine	0.690***	0.800	0.110***	0.698***	0.760***	0.620***
	(0.0179)	(0.0231)	(0.0260)	(0.0423)	(0.0287)	(0.0459)
Beer	0.529***	0.565	0.036***	0.427***	0.404***	−0.023***
	(0.0134)	(0.0110)	(0.0169)	(0.0196)	(0.0143)	(0.0287)
Sorghum	0.140***	0.117	−0.230***	−0.099***	0.171	0.270***
	(0.0399)	(0.0328)	(0.0510)	(0.0375)	(0.1532)	(0.1528)
Kakwani Index						
Total Alcohol Consumption	−0.073***	−0.056***	0.017***	−0.167***	−0.145***	0.220***
	(0.0238)	(0.0321)	(0.0152)	(0.0499)	(0.0346)	(0.0226)
Spirits	0.039	0.095	0.056***	- 0.0004	0.017	0.017
	(0.0329)	(0.0625)	(0.0193)	(0.0823)	(0.0575)	(0.0283)
Wine	0.038	0.143	0.105	0.034	0.117	0.083***
	(0.0387)	(0.0926)	(0.0243)	(0.0916)	(0.0830)	(0.0423)
Beer	−0.122***	−0.092**	0.030***	−0.237***	− 0.239***	−0.002
	(0.0327)	(0.0364)	(0.0180)	(0.0638)	(0.0380)	(0.0312)
Sorghum	−0.511	−0.540***	− 0.029	−0.764	− 0.472**	0.292***
	(0.0500)	(0.0451)	(0.0516)	(0.0605)	(0.2051)	(0.1536)

ªBootstrap SEs using 1000 resamples are reported in parenthesis. Significance levels are denoted as follows: ***1%, **5%, *10%

#### The Kakwani index of progressivity

Overall spending on alcohol beverages was regressive from 1995 to 2010/11 (Table 7). Expenditure on wine is consistently progressive over the years while expenditures on beer and sorghum were regressive over the years. Expenditure on spirits was progressive from 1995 to 2000, regressive in 2005/06 then progressive in 2010/11. The difference in Kakwani indexes between two-time periods (1995 and 2000; 2005/06 and 2010/11), indicates that spending on alcoholic beverages had a pro-poor ‘shift’ (less regressive) in progressivity between 1995 and 2000 and between 2005/06 and 2010/11. For instance, the Kakwani index for total alcohol beverages was estimated at − 0.073 in 1995 but it increased to − 0.056 in 2000; while in 2005/06 it was estimated at − 0.167 but increased to − 0.145 in 2010/11. This means that while, overall, spending on alcohol remains regressive, the changes over time (in the presence of significant increases in taxation on alcohol) show that this regressivity declined significantly.

The Kakwani index for spending on spirits and wine became more progressive (pro-poor ‘shift’) between 1995 and 2000, and between 2005/06 and 2010/11. Consumption expenditures on beer became less regressive (pro-poor ‘shift’) between 1995 and 2000 and slightly more regressive (pro-rich ‘shift’) between 2005/06 and 2010/11, although the changes were not statistically significant. Consumption expenditures on sorghum became more regressive (pro-rich ‘shift’) from 1995 to 2000 and less regressive (pro-poor ‘shift’) from 2005/06 to 2010/11.

## Discussion

This study shows that for both the structural and effective progressivity approaches, spending on alcoholic beverages is regressive; poorer households spend a significantly larger share of their total household consumption expenditure on alcohol than richer households. It became less regressive between 1995 and 2000; and between 2005/06 and 2010/11 (i.e. a pro-poor ‘shift’).

Based on the results in Table 7, one possible explanation for the pro-poor ‘shift’ in progressivity for spending on alcoholic beverages in South Africa is the reduction of inequality in consumption expenditure (a decrease in the Gini index). As noted before, changes in the Kakwani index of progressivity between two time periods (eq. 4) is driven by changes in the concentration index of spending on alcohol and changes in the Gini coefficient. The results show that there is no absolute change in the concentration index of spending on alcohol (*∆C* ≈ 0) between 2005/06 to 2010/11, while there is a decrease in the Gini index (*∆G* < 0) between 2005/06 and 2010/11. Therefore, since the concentration index has not changed between 2005/06 and 2010/11, the pro-poor ‘shift’ results from an improvement in the distribution of consumption expenditure.

The results for progressivity by type of alcoholic beverages show that expenditures on spirits, wine and beer- although not statistically significant, became more progressive (pro-poor ‘shift’); while, expenditures on sorghum became less regressive (pro-poor ‘shift’) between 2005/06 and 2010/11. This pro-poor ‘shift’ in expenditures on different alcoholic beverages between 2005/06 and 2010/11 resulted from a significant reduction in consumption expenditure inequality (*∆G* < 0) or an increased concentration in the consumption of alcoholic beverages among the rich. For example, for wine and sorghum, the pro-poor ‘shift’ resulted mainly from a positive change in the concentration index of spending on wine and sorghum (*∆C* > 0). The positive change in the concentration index may be explained by wine and sorghum spending being more concentrated among the richer households in 2010/11 than in 2005/06.

Results emerging from the progressivity analysis allow us to confirm alcohol consumption patterns seen in other alcohol literature sources [4, 6, 8]. For instance, Ataguba [6] found that poor households are more likely to consume sorghum beers while richer households are more likely to consume spirits and wines. In addition, our results confirm the assertion that alcohol consumption in South Africa differs by socio-economic status [6, 29, 30]. This difference in alcohol consumption pattern should be considered when alcohol policies are implemented given that South African alcohol consumers have different price elasticity of demand for different alcoholic beverages [8]. According to the National Treasury [8], spirits, malt beer and natural wine have price elasticity indicating that a 10% increase in price on spirits, malt beer and natural wine would decrease its demand by 7.5, 10.8 and 4.7%, respectively. Thus, although an increase in price may decrease the overall demand for alcoholic beverages, cross-price elasticity of alcoholic beverages may result in other shifts. For instance, an increase in price of spirits could lead to an increase in malt beer or natural wine consumption, while an increase in price for malt beer could increase the consumption of natural wine and vice versa. Sorghum is the only alcoholic beverage considered a “Giffen Good”, where its consumption increases with a price increase. These price and cross-price elasticities effects contribute to explaining the results in Table 5, for instance, the decrease in spending on spirts and beer, while there is an increase in spending on wine and sorghum in 2010/11.

The shift in spending on different alcohol beverages suggest that South African alcohol consumers are mitigating the effect of the increase in alcohol prices. It could also be due to the alcohol industry’s ability to reinvent itself. In South Africa, data from the South African Wine Industry Information & System (SAWIS) show that market share in volume decreased from 3.3% in 2006/2007 to 3.0% in 2010/11 for spirits and 8.2% in 2006/2007 to 7.5% in 2010/11 for wine; while it remained the same for unfortified wine (0.8%) and beer (79.2%). On the other hand, the ready to drink beverages (RTDs), such as alcoholic fruit beverages and spirit coolers, have substantially increased from 8.6% in 2006/07 to 9.5% in 2010/11. This increase in RTDs supports SAWIS’ idea that alcohol market in South Africa is ‘driven by innovation and new products’ to attract new consumers (especially young people and women). For instance, cross-sectional analyses using the Youth Risk Behaviour Survey (YRBS) show that adolescents (young people aged 10–19) in South Africa experienced an increase in binge drinking from 29.3% for males and 17.9% for females in 2003 to 33.5 and 23.7%, respectively in 2008; and a slight decrease to 30.3% for males and 20.1% for females in 2011 [31].

Although spending on alcohol beverages has been decreasing (Table 1), alcohol consumption in South Africa has increased over recent years [10]. Data from SAWIS show that alcohol volume increased by 5.7% (3.5 billion to 3.7 billion liters) between 2006/07 and 2010/11 [32]. The results of the study demonstrated that the key driver of progressivity of spending on alcoholic beverages between 2005/06 and 2010/11 was a result of a more equal distribution of consumption expenditure. Thus, the fact that the concentration index of spending on alcohol has not been a major player in increasing progressivity means that there is an opportunity to increase progressivity using alcohol policies. If alcohol policy can reduce the absolute change in concentration indexes sufficient to offset the absolute change in the consumption expenditure inequality, then, following eq. 4, this change in the alcohol concentration index will positively impact the Kakwani index of progressivity creating a further pro-poor ‘shift’.

Based on international evidence, there are other policy options, besides increasing alcohol taxes, to decrease alcohol-related harms [33, 34]. Examples of other cost-efficient policies include age restrictions on the sale of alcohol, reduced access to retail outlets, a comprehensive advertising ban, enhanced enforcement of on-premises policies and legislation, and interventions with at-risk drinkers. A combination of these policies is advocated. Currently, there are two alcohol policies under consideration by the South African government. First, the Department of Trade and Industry is proposing changes to the National Liquor Act to impose further restrictions on alcohol sales, increase the minimum drinking age from 18 to 21 years and ban the sale of alcohol in proximity to schools and places of worship [35]. The second is the Control of Marketing of Alcohol Beverages Bill proposed by the Department of Health, which, along with other restrictions, aims to ban any alcohol advertising in shops, media, radio, sports events, but does not ban advertising at the point of sale such as a *shebeen*, liquor store or bar. Neither bill address alcohol pricing nor taxation as a method of addressing alcohol-related harms. While pricing policies are effective, policymakers should exercise caution when applying them as the alcohol market is highly complex, thus monitoring pricing changes is advocated. Additionally, when using price policies, consideration should also be given to inequities behind alcohol consumption, such as who are more exposed and vulnerable to alcohol harms [36]. For future research, and as the policy space changes, impact evaluation methods could be used to assess the impact of alcohol policy on alcohol consumption by different population groups, including socioeconomic groups.

One of the study’s strengths is that alcohol spending progressivity was assessed using comparable nationally representative data. In addition, this analysis provides an initial attempt to assess progressivity of alcohol expenditure at the household level using two different progressivity approaches, structural and effective progressivity. A study limitation is that alcohol expenditures do not account for homemade alcohol production (called unrecorded alcohol). Homemade or informally produced alcohol beverages remain a big part of South African culture. According to the World Health Organization [1], in 2010 unrecorded per capita alcohol consumption (APC) in South Africa was estimated at 2.9 l in pure alcohol (26.4% of total APC). Thus, this study potentially underestimates alcohol spending for the poorest households since they are more likely to consume homemade or informally produced alcohol beverages [8, 36]. Another limitation is that there is a possibility that alcohol consumption and expenditure variables are underestimated due to recall bias for the 1995 and 2000 datasets. To minimize any potential bias, the 1995 and 2000 data, that were considered similar, were compared.

## Conclusion

Spending on alcoholic beverages in South Africa remained regressive, albeit declining, between 1995 and 2010/11. This is because the fraction of consumption expenditure spent on alcoholic beverages remained higher for poorer households compared to richer households. Based on the results, there is an opportunity to further reduce the regressivity using coherent alcohol policies. For instance, if the South African government continues its gradual increase in alcohol taxation rates, this could continue to have a pro-poor effect on progressivity. However, price elasticities and substitution effects must be taken into consideration and trends monitored (e.g. pricing changes, consumption and spending patterns). Although the results presented in this paper are based on quantitative analysis, there is a need for further research, especially using qualitative methods, to unpack why alcohol spending became less regressive over the years. This must go beyond just looking at changes in the distribution of alcohol expenditure. In addition, further research needs to address the distribution of alcohol-related harm and the effects of alcohol consumption on health so that policymakers can implement additional alcohol policies to track consumption patterns and decrease alcohol-related harms.

## Data Availability

The datasets generated during and/or analysed during the current study are available in the Statistics South Africa repository, https://www.datafirst.uct.ac.za/

## Abbreviations

APC: Per capita alcohol consumption
C: Concentration index
CPI: Consumer Price Index
DALYs: Disability-adjusted life years lost
G: Gini index
IES: Income and Expenditure Survey (IES)
K: Kakwani index
RTDs: Ready to drink beverages
SAWIS: South African Wine Industry Information & System
SES: Socio-economic status
YRBS: Youth Risk Behaviour Survey

## Footnotes

1
https://www.datafirst.uct.ac.za/
